# An interdisciplinary assessment of private conservation areas in the Western United States

**DOI:** 10.1007/s13280-020-01323-x

**Published:** 2020-02-21

**Authors:** Cristina Quintas-Soriano, Dainee M. Gibson, Jodi S. Brandt, María D. López-Rodríguez, Javier Cabello, Pedro A. Aguilera, Antonio J. Castro

**Affiliations:** 1 Social-Ecological Interactions in Agricultural Systems, Faculty of Organic Agricultural Sciences, University of Kassel, 37213 Witzenhausen, Germany; 2 Social-Ecological Research Lab, Department of Biological Sciences, Idaho State University, 921 South 8th Avenue, Pocatello, ID 83209 USA; 3 Departamento de Biología Vegetal y Ecología, Centro Andaluz para la Evaluación y Seguimiento de Cambio Global (CASCG), Universidad de Almería, La Cañada de San Urbano, 04120 Almería, Spain; 4 Human–Environment Systems Center, Boise State University, Boise, ID 83725 USA; 5 Internet Interdisciplinary Institute (IN3)-Universitat Oberta de Catalunya (UOC), Av. Friedrich Gauss 5, 08860 Castelldefels, Barcelona Spain; 6 Informatics and Environmental Research Group, Department of Biology and Geology, University of Almería, Almería, Spain

**Keywords:** Conservation bundles, Ecosystem services, Private lands, Protected areas, Social-ecological systems, Transdisciplinary science

## Abstract

Conservation easements are the fastest growing private conservation strategy in the United States. However, mechanisms to assess private land conservation as well as their support by the general public are not well understood. This study uses the ecosystem services framework for assessing existing private lands in Idaho and identifies areas for future conservation easements. Using conservation targets of the land trust as a guide for selecting ecosystem services, we (a) mapped the spatial delivery of conservation targets across public and private lands, (b) explored public awareness in terms of social importance and vulnerability, and (c) mapped future priority areas by characterizing conservation bundles. We found that public lands provided the highest levels of conservation targets, and we found no difference in conservation target provision between private areas and conservation easements. The spatial characterization of conservation target bundles identified potential future priority areas for conservation easements, which can guide planning of land trust conservation efforts.

**Electronic supplementary material:**

The online version of this article (10.1007/s13280-020-01323-x) contains supplementary material, which is available to authorized users.

## Introduction

The Convention of Biological Diversity (CBD 1992) urges to establish a system of protected areas for the in situ preservation of global biodiversity and maintenance of ecosystem services (ES, defined as the benefits that humans obtain from ecosystems; MEA 2005). Consequently, there is a political goal to integrate 17% of the land surface into a global protected area network by 2020 (CBD 2010). Due to limited resources availability for establishing new protected areas, countries are required to design and implement complementary area-based conservation policies (CBD 2010). In this sense, governments are encouraged to cooperate with private initiatives in developing methods for promoting conservation strategies in collaboration with local agencies and NGOs.

In recent decades, the implementation of conservation strategies on private lands, hereafter private conservation areas (Pasquini et al. 2010), is increasingly being recognized as a strategy to complement current protected areas networks (Cortes-Campano et al. 2019). Currently, private conservation areas protect several million hectares of natural habitat and cultural landscapes across the world (e.g., Jones et al. 2005; Sims‐Castley et al. 2005). These new private conservation efforts are commonly implemented by practitioners as a strategy to deliver benefits to society that contribute to social‐welfare goals, for instance, through job creation where the land is managed for recreational activities and other profitable business (Chacon 2005; Rambaldi et al. 2005). However, despite the interest of many countries to develop new conservation initiatives, the contribution of these areas to the preservation of biodiversity and ES is difficult to assess. Hence, new methodological approaches are required to further understand the contribution of these spaces to global conservation targets (Merenlender et al. 2004; Pasquini et al. 2010).

In the United States (U.S.), conservation easements stand out as the fastest growing private conservation strategy (Dayer et al. 2016). Conservation easements are legally binding, voluntary conservation agreements on private lands that do not transfer ownership of the land, but define limitations to future development or management rights (Rissman 2013; Quinn and Wood 2017). The majority of conservation easements are promoted by local and state land trusts, which are non-governmental organizations that conserve land by negotiating and/or purchasing land in order to preserve it for natural, historical, personal, or economic values (Stolton et al. 2014; Peters et al. 2017). Over the last 30 years, the number of conservation easements on private land in the United States has increased exponentially in order to protect natural and agricultural resources (Merelinder et al. 2004; Stolton et al. 2014). Currently, over 1700 land trusts are conserving more than 19 million hectares in the U.S. (Peters et al. 2017).

Since land trusts are generally small organizations that act independently, each land trust and individual conservation easement has its own conservation targets. For example, different easements in Virginia and North Carolina have different management priorities when it comes to bird watching and water purification (Villamagna et al. 2015). In the western U.S., public protected areas protect more land than private protected areas, but private land conservation via conservation easements has become a popular alternative to underfunded and controversial public acquisition techniques (Brunson and Huntsinger 2008). Especially as large tracts of public protected areas have been downgraded or sold, the value of conservation easements has become more visible in the region (Defries et al. 2007; Villamagna et al. 2015). These conservation easements have emerged as a means to save traditional ranching culture, protect the landscape from exurban subdivisions, preserve open space, safeguard rangeland ecosystems, and maintain the cultural heritage of ranching (Brunson and Huntsinger 2008). Therefore, conservation easements protect valuable benefits of the landscape while allowing traditional use of the land. Under the law, conservation easements protect the land in perpetuity. To ensure that the legal framework for land conservation will endure, all land trusts are committed to building strong public support for land conservation (Stolton et al. 2014). Furthermore, despite the increase in conservation easements in land conservation, the public remains largely unaware of this private land approach to conservation (Villamagna et al. 2017). Implementing on-the-ground conservation actions on private land mostly depends on landowners’ willingness to collaborate with conservation agencies and their management capabilities (Bastian et al. 2017; Vizek and Nielsen-Pincus 2017; Cortes-Campano et al. 2019).

The ES framework provides several tools that can be used to advance in the assessment of private conservation areas. For instance, the bundle analyses distinguish groups of ES (i.e., conservation targets) that are produced on the landscape with similar provision levels, and the different bundles can characterize the range of opportunities for future conservation areas (Raudsepp-Hearne et al. 2010; Queiroz et al. 2015; Quintas-Soriano et al. 2019). However, the major challenge for operationalizing ES bundle analyses is to integrate public perceptions and preferences with conservation goals. Here, we propose an interdisciplinary methodological approach for assessing existing conservation easements of a regional land trust in Idaho, U.S. First, we quantify and map the spatial provision of the five conservation targets and compare across public protected areas, private lands, and existing conservation easements. Second, we explore public perceptions regarding conservation targets in terms of social importance and vulnerability. Third, we identify additional private areas where conservation targets are preserved and socially supported. Finally, we discuss the potential for these areas to be declared as future private conservation areas, and the implications of this approach for conservation in other regions.

## Materials and Methods

### Study area: The Portneuf River watershed and the Sagebrush Steppe Land Trust

**Fig. 1:**
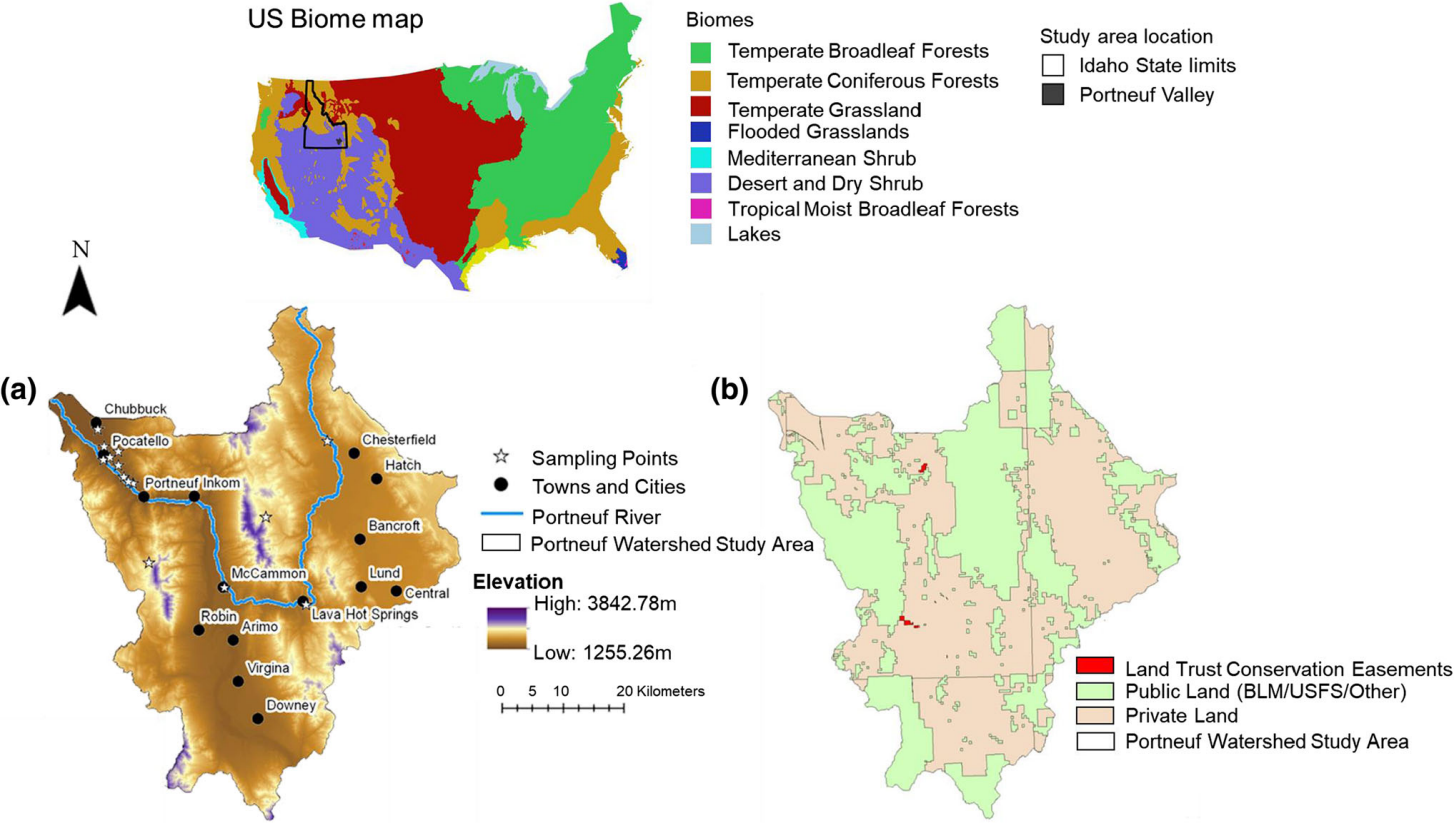
U.S. biomes map and location of Idaho state and the study area. Portneuf watershed in SE Idaho with elevation and social sampling locations (**a**). Locations of Sagebrush Steppe Land Trust conservation easements, public protected areas (including Bureau of Land Management and Forest Service lands), and private lands within the Portneuf watershed (**b**)

The Portneuf River watershed is located in SE Idaho, U.S. (Fig. 1a). This region has a semi-arid climate of hot and dry summers and moderately long winters. Rangeland covers about 55.6% of the total watershed, cropland covers approximately 22.3%, forest consists of approximately 17% of the watershed area in higher elevations, and urban areas consist of 4.2%. The urban land and crop agriculture is located in the lower, flat elevations in the watershed, while the grazing occurs in mid to high elevations, along with some in the flat valleys in the watershed (Fig. 1b). About 34% of the land in the Portneuf watershed is contained within protected areas, with the majority being in public protected areas (13% managed by the Bureau of Land Management (BLM) and 21% by the U.S. Forest Service (USFS) (Fig. 1b). Private lands (65% of the study area) are destinated to agricultural lands, farming activities, farm-related businesses, and agricultural uses in the community (Fig. 1b).

The Sagebrush Steppe Land Trust is the local land trust whose service area includes the Portneuf Valley (Fig. 1b). Currently 0.69% of the Portneuf River watershed is protected through conservation easements, and the Sagebrush Steppe Land Trust is currently prioritizing the creation of new conservation easements in the valley. This corresponds with the two conservation easements that are in the study area that cover approximately 600 hectares.

### Quantifying and mapping provision of conservation targets

Our methodological approach is based on the correspondence between conservation targets and ES. Thus, the Sagebrush Steppe Land Trust conservation targets were translated into ES (Table 1). The five conservation targets maps were created on a 30 × 30 m grid (Appendix S1). Due to the fact that the average conservation easement size in the Portneuf watershed corresponds approximately to 1 km^2^, to preserve heterogeneity in the data while maintaining a dataset that was manageable for computation, we scaled up the resolution to a 1 × 1 km grid. Then, to compare all conservation targets, we standardized them using the raster calculator in ArcMap 10.5. We used minimum–maximum normalization to standardize the conservation target on a 0 to 1 scale following Castro et al. (2015). Because this normalization technique is sensitive to outlier, minimum, and maximum values, the values within the conservation target maps that occur outside the 5th or 95th percentile were assigned the 5th or 95th value, respectively (Castro et al. 2015). Then, we combined and normalized all conservation targets to represent areas with the highest and lowest levels of all conservation targets' provision in a single area. Finally, we compared conservation target provision within the public protected areas, private lands, and existing land trust conservation easements and explored differences using a non-parametric Mann–Whitney U test. All of the statistical analyses were performed using R 3.3.1 (R Core Team).

**Table 1:** Sagebrush Steppe Land Trust conservation target correspondence with ecosystem services, method of mapping, and social survey wording. The land trust conservation target, ecosystem service, model or proxy for mapping, and way of wording the ecosystem service within the social perception survey panel that provided ecosystem services, called “contributions from nature to people” for survey respondents

Land trust conservation target	Ecosystem service	Method of mapping	Wording in social survey panel
Critical wildlife habitat	Habitat quality	InVEST habitat quality model	“Habitat for species”
Water quality	Water quality	EnviroAtlas total stream impairment length	“Water Quality”
Open space and scenic views	Scenic quality	InVEST scenic quality model	Not included
Recognized historic and cultural value	Cultural heritage	Historic land-use trends weighted by survey data	“Cultural heritage”
Recreational Access	Recreation	Density of trails	“Recreation/ecotourism”

### Public awareness regarding conservation targets

#### Questionnaire design and social sampling strategy

In the summer of 2016, we conducted a social sampling using face-to-face questionnaires in the Portneuf watershed. Overall, 471 valid surveys were completed (Table 2; Appendix S2). Sampling occurred at 30 distinct locations in the Portneuf Valley (Fig. 1a), with a focused effort on populated and tourist areas within the study site. The population sampled was a convenience sample (Quintas-Soriano et al. 2018; Narducci et al. 2019). The questionnaires collected information regarding local perceptions towards ES, perceived impact of land use/land cover on local ES, and sociodemographic information (see Appendix S2).

**Table 2:** Sociodemographic characterization of the Portneuf River Watershed social sample

Categories	% of respondents
Gender	
Female	45
Male	55
Age	
< 25 years	23
25–39 years	32
40–54 years	19
> 55 years	25
Income	
< $19 999	14
$20 000–$39 999	14
$40 000–$59 999	15
$60 000–$79 999	10
> $80 000	19
Educational level	
Less than high school	2
High school degree	14
University/college	82
Sense of place	
City/county	6
Southeastern Idaho	30
Idaho	23
Western USA	21
USA	6
Ethnic background	
White	70
Black, African-American	8
Native-American	3
Asian American	2
Latino or Hispanic	10
Multi-racial	1
Other	4
Membership in an environmental association	
Yes	9
No	91

#### Social perceptions of conservation targets

Social perceptions of local respondents were explored to evaluate public awareness of conservation targets. We began with a free-listing technique in which respondents were asked to name all of the possible benefits they could think of that the ecosystems in the study area provide (Quintas-Soriano et al. 2018). Those examples provided were coded into ES following the international ES classification of CICES (www.cices.eu; Haines-Young and Postchin 2013). Ambiguous responses and those that could not be categorized into any ES were excluded. Following Martín-López et al. (2012), we developed initial categories for each example of a benefit from the watershed. Then, we grouped similar responses of a given category into groups that corresponded to an established ES. We then estimated the percentage of respondents in each location who listed specific services. From this grouping, we estimated difference in public awareness for conservation targets using a *χ*^2^ test in R 3.3.1 (R Core Team).

To assess conservation target vulnerability, we compared the trend that survey respondents believed that conservation targets underwent in the last 10 years (Quintas-Soriano et al. 2014, 2016; Castro et al. 2016). The conservation targets on the ES panel included cultural heritage, habitat quality, recreation, and water quality (Appendix S3). Scenic quality was not included in our ES panel, so our analysis of vulnerability could not include this goal. All survey respondents were asked to indicate the perceived trend (i.e., decreasing, stable, or increasing) of ES over the past 10 years. We then estimated the percentage of respondents who listed vulnerability types for specific services and we analyzed the differences in service vulnerability perceptions between the four conservation targets using a *χ*^2^ test in R 3.3.1 (R Core Team).

### Characterizing and mapping alternative private areas for conservation

All conservation target maps were standardized from 0 to 1 and established with a resolution of 5 × 5 km grids to be applicable for land trust management (Appendix S1). Then we created maps of the residuals from the average conservation target provision in the watershed (Quintas-Soriano et al. 2019). These residuals represent “hot” (higher provision) and “cold” (lower provision) spots of 5 × 5 km areas (Queiroz et al. 2015).

Conservation target bundles are here defined as portions of private lands where multiple conservation targets are simultaneously provided. Since the land trust only works with private landowners to create conservation easements, we limited our analysis of private conservation areas to private lands in the Portneuf watershed (Fig. 1b). We used cluster analysis to identify 5 × 5 km areas in private land with similar provision levels of conservation targets (Quintas-Soriano et al. 2019). K-means clustering was used to identify five distinct conservation target bundles in the Portneuf watershed. The selection of the number of clusters was based on cluster robustness and knowledge of the area (Raudsepp-Hearne et al. 2010; Queiroz et al. 2015). We used flower diagrams to visualize conservation target bundles. These bundles were calculated from the normalized values of each conservation target map, and the size of the flower petals represents conservation target provision. Each conservation target bundle represents a different opportunity for future private conservation areas because they provide the goals of the land trust in different quantities. In addition, interactions among conservation targets were analyzed using a principal component analysis (PCA) (Queiroz et al. 2015). PCA identified the main explanatory factors of the variability and distribution of the conservation targets across the watershed. We used QGIS 2.12.1 to map the spatial distribution of conservation targets and R to conduct all spatial analysis and produce all the figures (R Core Team 2018).

## Results

### Conservation target provision in the Portneuf River watershed

**Fig. 2:**
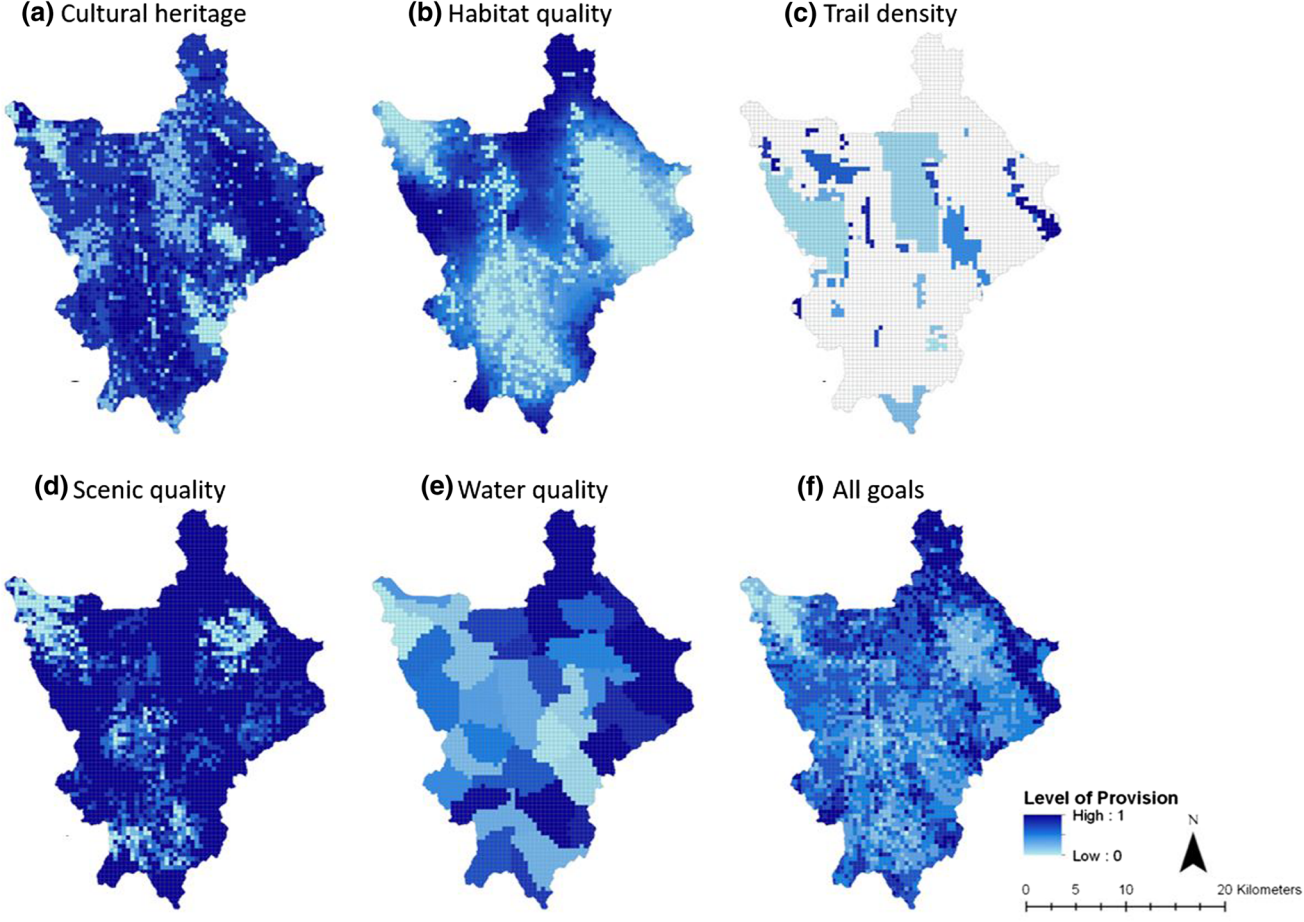
Conservation target standardized provision in the Portneuf River watershed and map of the combination of the five conservation targets. Light blue indicates minimum provision while dark blue indicates maximum provision of each conservation goal

The spatial distribution of the conservation target provision varied throughout the Portneuf watershed (Fig. 2; Appendix S1). Cultural heritage was highest in the southern and eastern parts of the watershed. The lowest provision of cultural heritage occurred in the central and northwestern regions of the watershed corresponding with areas with influence from urban and exurban land uses, as well as in higher elevation regions in the watershed (Fig. 2a). High values of cultural heritage were mostly concentrated in agricultural and rangeland areas, which are dominated by agricultural production, grazing, and sagebrush-steppe vegetation. Values of cultural heritage tend to occur in mostly flat areas of lower elevation, as this conservation target is tied closely with the crop and cattle agriculture that occurs in these areas.

Highest values of habitat quality were found in public lands dominated by natural forests and rangeland (Fig. 2b). These areas are in higher elevation, most often located in the public lands of the watershed. Vegetation is marked by sagebrush-steppe species and conifer forests. The major areas of low habitat quality included the urban center of Pocatello in the northwestern corner of the watershed, the southern agricultural valley, and the eastern agricultural valley in the Portneuf watershed. Low habitat quality occurred in town and urban centers as well as in agricultural lands. Therefore, lower elevation and flatter slopes tended to have lower habitat quality in the Portneuf watershed because this is where agricultural and urban areas occur.

Trail density was mainly present in areas dominated by sagebrush-steppe vegetation and conifer forests. The southern part of the watershed tended to have lower trail density, which is influenced by fewer large population towns in this region than in the northern part of the watershed (Figs. 1a, 2c).

Scenic quality was largely impacted by natural vegetation and altitude, including sagebrush-steppe species, deciduous forests, and conifer forests. Locations in the watershed that had views of these natural flora and the mountains were marked by higher scenic quality, such as the central ridgeline across the Portneuf watershed and the higher elevations on the boundary of the watershed (Figs. 1b, 2d). On the other hand, areas of higher elevation that surround heavy industrial and agricultural land uses had lowest scenic quality, which is best demonstrated through the low scenic quality on the hills surrounding the industrial railroad and phosphate mine in the northwestern-most corner of the Portneuf watershed. Lower elevation areas (less than 1600 m) that cannot view the heavy anthropogenic land uses had medium levels of scenic quality because the view of a positive or negative impact on view influences the InVEST scenic quality model, as demonstrated by central region of the watershed (Figs. 1a, 2d).

The distribution of water quality varied significantly in the watershed (Fig. 2e). Highest values of water quality were found in the northeastern third of the watershed where the upper portion of the Portneuf River is located. Furthermore, areas in the northeastern part of the watershed, marked by higher elevation and sloping above the Portneuf River, had high water quality. Conversely, regions dominated by agricultural and urban land use, especially those in the lower portion of the Portneuf River, tended to have lower water quality (Fig. 2e). As more of the water from the river is diverted for agricultural and urban uses, the water quality decreased (Fig. 1a). These regions included the urban center of Pocatello in the northwestern corner, the southern agricultural valley, and the eastern agricultural valley in the Portneuf watershed. These regions are of lower elevation (less than 1700 m) and are flatter, which supports urban and agricultural land uses and allowed more impairments to reach the water bodies in the region (Figs. 1a, 2e). These impairments thus reduced water quality because the relative amount of impaired stream length increases in these lower quality regions.

The resulting map of all conservation targets showed highest conservation target provision in the northeastern portion of the watershed (Fig. 2f). The mid-level elevation locations in and near public lands also had some of the highest levels of all conservation target provision in the Portneuf watershed. Urban and exurban areas had the lowest levels of all conservation target provision (0.0–0.3; Fig. 2f). The medium levels of all conservation target provision (0.31–0.6) occurred in the agriculturally dominated, flat valleys of the watershed (Figs. 1a, 2).

**Fig. 3:**
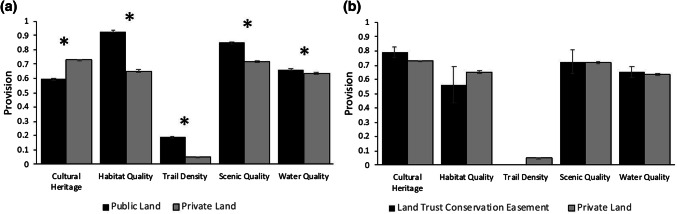
Public land versus private land level of provision of conservation target (**a**) and land trust conservation easements versus private land provision of conservation targets (**b**). Bars represent the average provision of that conservation goal for each area, while bars indicate the standard error of the mean. Statistical significant differences (*p* < 0.001) as determined by Mann–Whitney *U* test between level of provision is denoted by an asterisk (*)

Public lands provided, on average, higher levels of habitat quality, trail density, scenic quality, and water quality than private lands (Fig. 3a). On the other hand, private areas provided higher levels of cultural heritage (0.73 ± 0.006). Land trust conservation easements provided similar levels of conservation targets as the private areas in the Portneuf watershed (Mann–Whitney *U* test, *p* > 0.05, Fig. 3b).

### Public awareness and perceived vulnerability of conservation targets

**Fig. 4:**
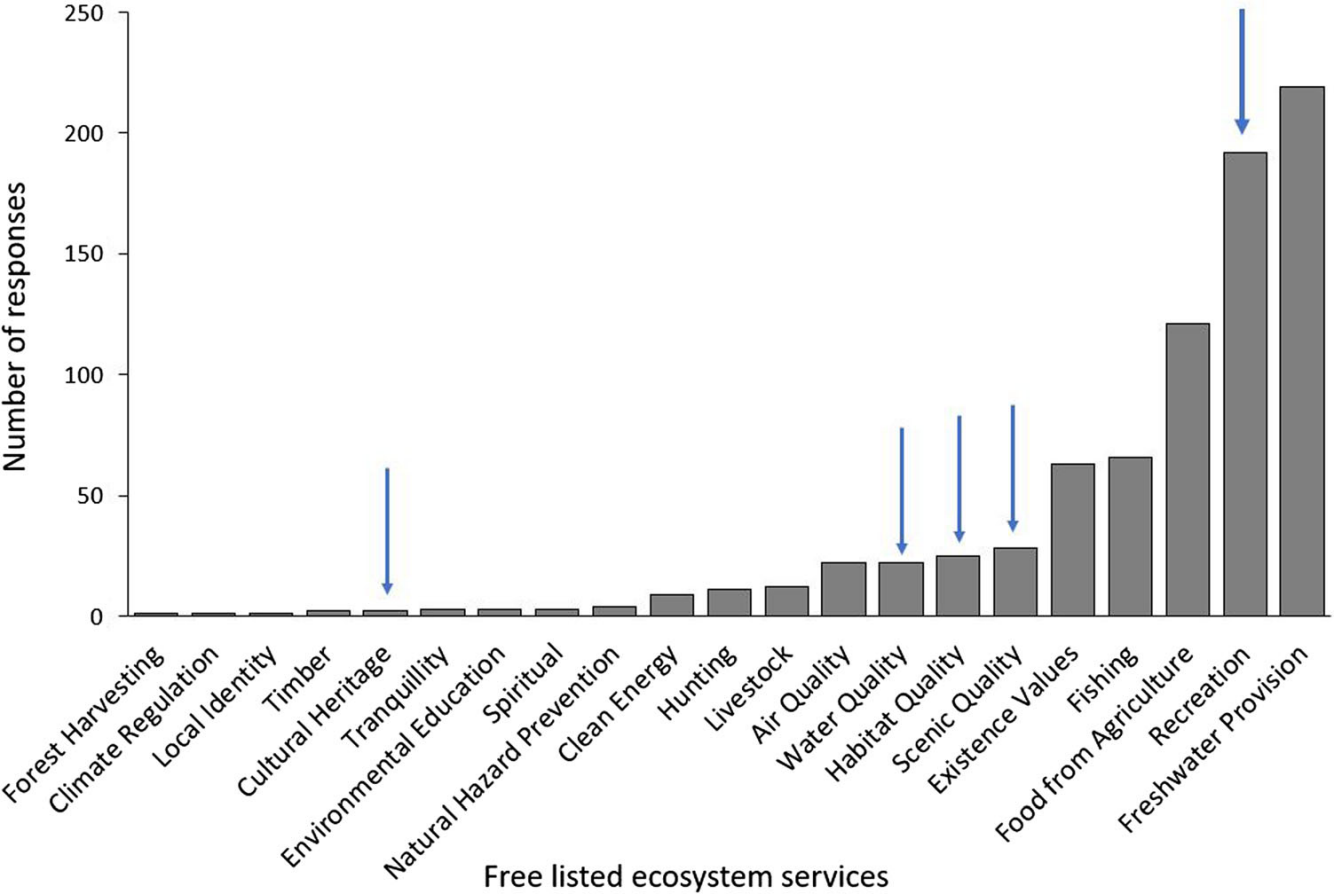
Perceived awareness of local ecosystem services. The public awareness of land trust conservation targets is indicated by a blue arrow

We found significant differences between the public awareness towards conservation targets (i.e., ES) in the watershed (*χ*^2^ test, *p* > 0.05, Fig. 4). Respondents recognized as most important services freshwater provision, followed by recreation, food from agriculture, fishing, and existence values (Fig. 4). The most mentioned free-listed services corresponded with four out of the five conservation targets. Recreation and scenic quality were the most visible conservation targets, while water and habitat quality have a lower visibility to respondents. Finally, cultural heritage had low public awareness, with only two respondents identifying cultural heritage as an unprompted benefit from the Portneuf watershed.

**Fig. 5:**
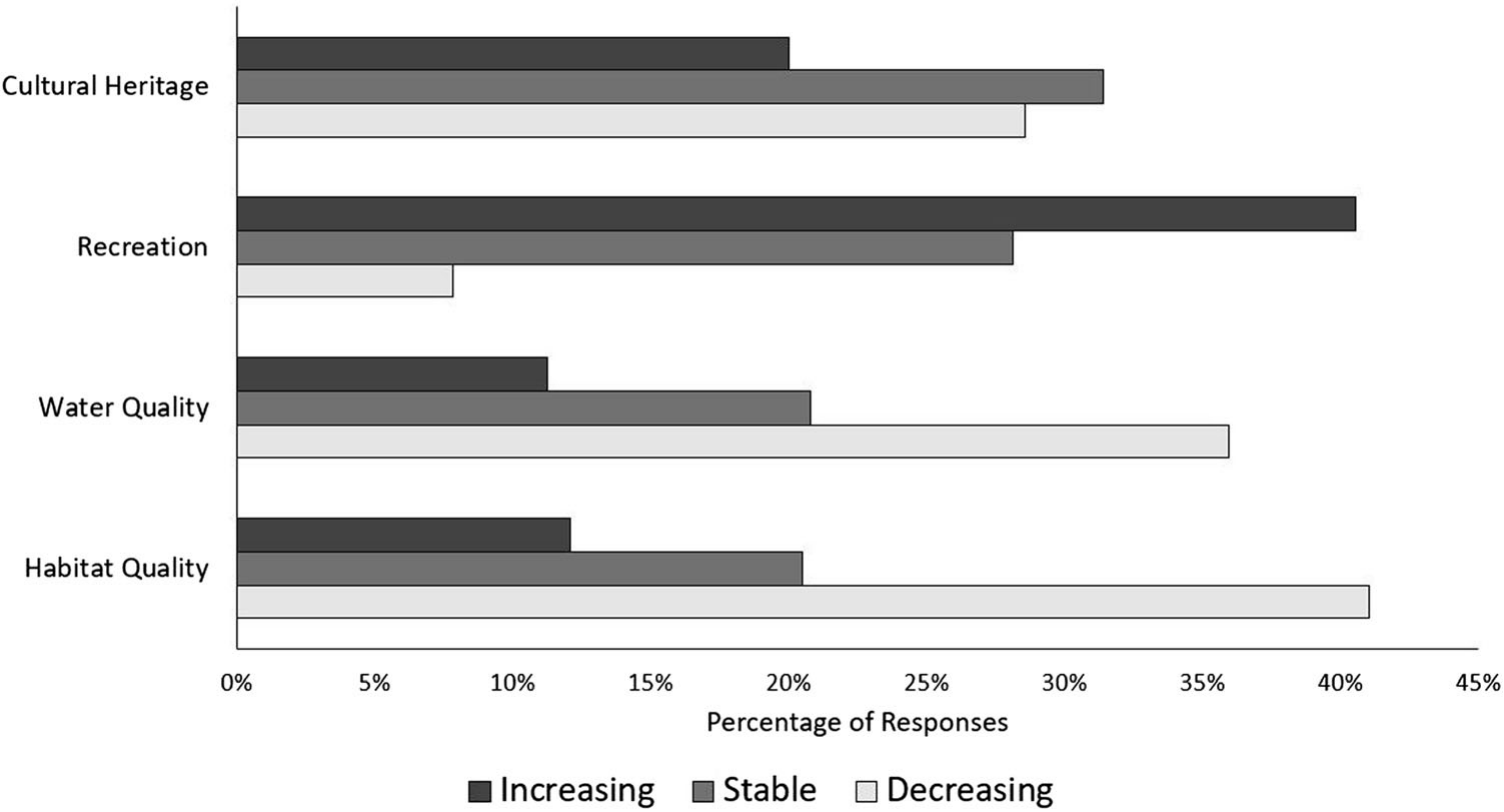
Perceived vulnerability by locals regarding land trust conservation targets

We found differences in perceived vulnerability among the four conservation targets (*χ*^2^ test, *p* < 0.001, Fig. 5). Water quality and habitat for species were most perceived by the public as being in decline over the past 10 years. Recreation was perceived as increasing in the Portneuf watershed during the past 10 years. Finally, cultural heritage was perceived as being mostly stable, with some perceptions of decline, over the past 10 years.

### Identification of alternative private conservation areas through hot and cold spots and bundles of conservation targets

**Fig. 6:**
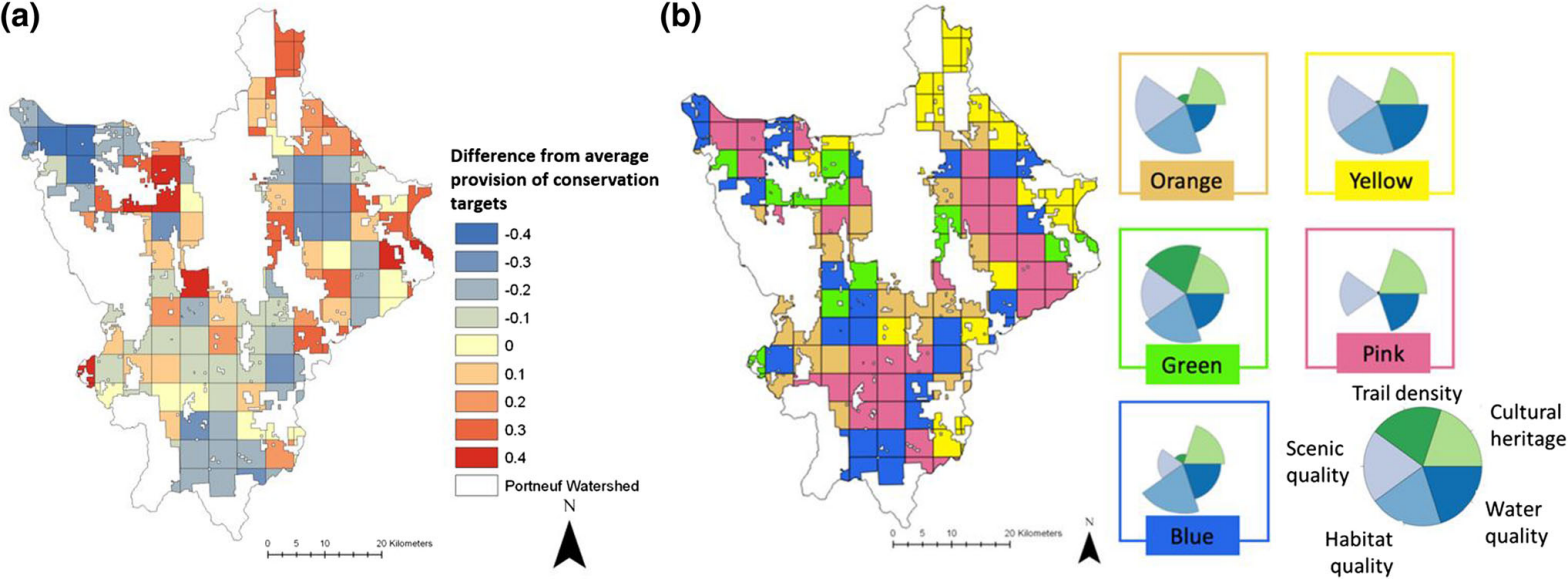
Hot and cold spots and bundle analysis for conservation targets across the study area. **a** Hot spots (represented by an increasing gradient of red) are areas with particularly high provision of land trust conservation targets, while cold spots (represented by a decreasing gradient of blue) are areas where the provision of land trust conservation targets is particularly low. **b** Bundles of conservation targets identified by k-means clustering for private land in the study area. The five groups of bundles (on the right side of the figure) are represented by rose-wind diagrams. The color of the box around each rose-wind diagram corresponds with the area of the map that is the same color. The flower diagrams are dimensionless, as they are based on normalized data for each service, and a higher surface area of a petal indicates the higher provision of a particular conservation target

Within private land, the hot spots of conservation targets occurred around the borders of public lands and in the northern half of the watershed (Fig. 6a). The cold spots of conservation targets occurred around the urban center of the City of Pocatello in the northwest corner of the watershed and in the agricultural centers in the east and south of the watershed. Hot spots are areas with particularly high provision of land trust conservation targets, while cold spots are areas where the provision of land trust targets are particularly low.

The PCA analysis identified two gradients that explained the variations of conservation targets across the study area (Appendix S4). The first PCA component explained 31% of the variance and corresponds to a gradient of human impact and elevation. The second PCA component, 23% of the variance, corresponds to a gradient of population density. Cluster analysis defined five groups of bundles of conservation targets within the private land of the Portneuf watershed (Fig. 6b). The orange bundle grouped high level of supply of scenic quality, habitat quality, and cultural heritage. These areas occurred between areas dominated by agricultural production and the public lands in the Portneuf watershed and provided lower levels of water quality and greatly reduced levels of trail density. The yellow bundle corresponded with highest levels of scenic quality, habitat quality, and water quality. These areas occurred in agriculture-dominated areas on the border of public protected areas and provided some cultural heritage with greatly reduced levels of trail density. In the green bundle, all conservation targets were provided in high levels. This bundle occurred in areas with higher elevation on the boundaries of public protected areas in the Portneuf watershed. The pink bundle presented high levels of scenic quality, water quality, and cultural heritage. This bundle occurred in areas dominated by agricultural production, which had the lowest levels of habitat quality and trail density. Finally, the blue bundle corresponded with highest provision of habitat quality and occurred along the lower elevation boundaries of public lands. These locations did provide some capacity to preserve water quality and cultural heritage, with low levels of scenic quality and greatly reduced levels of trail density.

## Discussion

This study implements a methodological approach to assess conservation easements in order to better understand the role of private land conservation strategies in the Western U.S. While the assessment of the role of protected areas to maintain well-being is commonly studied (Palomo et al. 2013, 2014), the role of conservation easements remains less investigated (Villamagna et al. 2013, 2015). Our interdisciplinary method showed that the conservation targets of the Sagebrush Steppe Land Trust are differently distributed throughout the Portneuf watershed. The five conservation goals showed different supply distribution across the study area, although in specific regions of the study area we found high patters of supply that allowed us to identify future areas for conservation. Additionally, the analysis of social importance shows that the public recognizes as important four of the five conservation targets, which socially support the implementation of futures conservation easements. Finally, the bundle analysis identified alternative areas (see Fig. 6b, green bundle) where all conservation targets are preserved, which might guide conservation efforts towards future private conservation areas (Villamanga et al. 2013).

Because conservation easements are the fastest growing private land conservation strategy in the U.S., our findings have important policy implications to make operative the ES approach in private conservation initiatives (Dayer et al. 2016). Currently, over 19 million hectares of land is under easement in the U.S. and millions of dollars annually are invested in new easements (Peters et al. 2017; Quinn and Wood 2017). However, mechanisms to assess conservation easements are not well established. Our study demonstrates that the application of ES can provide insights for the interdisciplinary assessment land trust conservation. Since land management policy in the U.S. is strongly influenced by the opinion, preferences and demands of the public, incorporating the opinion of local residents result essential to promote a more cost-effective and public-supported organization (Villamagna et al. 2013, 2017; Palomo et al. 2014). Additionally, by identifying spatially explicit bundles where multiple conservation targets occur, the land trust can target specific parcels and private landowners where to prioritize conservation efforts. We suggest incorporating spatial prioritization information such as what we produced to the network of landowners as a strategy to promote the goals and mission of the land trust in the region (Villamagna et al. 2015).

The private conservation areas identified in our analysis should be interpreted within the context of the limitations of our methodological approach. First, our approach differs from more traditional, smaller-scale ecological studies in that our study is a landscape scale, interdisciplinary assessment of different conservation targets (i.e., ES), in which we compare the potential capacity of different land units to preserve conservation targets. We assumed linearity between land trust conservation targets and the model and proxies used to quantify the Portneuf landscapes’ capacity to deliver them, which is common in the assessment of ES (Castro et al. 2013; Quintas-Soriano et al. 2014). For instance, we used trail density as a proxy to evaluate recreation. This estimation could be more accurate if we had more detailed information on the annual number of visitors recreating on Portneuf trails, which would allow us to accurately estimate the environmental pressure that recreation places on public and private lands. Egoh et al. (2012) reviewed this issue and stated that while provisioning services can be directly quantified, most cultural services are less straightforward, and researchers must rely on indicators or proxy data for their quantification. Our study advances this topic by using two new proxies for mapping recreation and cultural heritage, two of the services considered more difficult to quantify (Plieninger et al. 2013). Second, assessing the public awareness of conservation targets through spatially explicit exercises is challenging (Brown and Kytta 2014). While our study does not map social perceptions regarding conservation targets, our social assessment is spatially explored across particular landscapes (Fig. 1a), which may inform the Sagebrush Steppe Land Trust on the degree of acceptance that local communities may have for specific conservation targets. Third, the spatial scale used in the bundle analysis of conservation targets continues to be a subject of debate (Spake et al. 2017) because there can be a loss of accuracy as a result of the normalization and standardization of ES maps (Quintas-Soriano et al. 2019). Most ES bundle studies have used the municipality level, with the justification being that it is the smallest administrative scale that decisions are made (see for example Raudsepp-Hearne et al. 2010; Renard et al. 2015), and thus this scale enables connection with land managers and decision makers (Queiroz et al. 2015). In addition, while a larger scale might facilitate the visualization of the ES bundles, there would be limitations associated with the quality of data (Carpenter et al. 2009; Martín-López et al. 2012; Castro et al. 2014). In our study, we developed a grid bundle analysis to improve the spatial resolution and provide results at a spatial resolution that is relevant for decision-making for the Sagebrush Steppe Land Trust. Deciding the appropriate resolution in ES bundle analysis is a key step in deriving accessible results that will inform land trust land management strategies. Therefore, the bundle resolution must be agreed upon by scientists and decision makers in order to ensure its application. In our study, the selection of a 5 × 5 km size grid for the bundle analysis was based on two premises: (1) our ability to preserve heterogeneity in the original data while maintaining a dataset that is manageable for computation; and (2) identify areas that have a higher likelihood of containing lands with more than one landowner in the grid cell, which increases the number of potential landowners per grid.

Conservation initiatives on privately owned land can help to mitigate the loss of global biodiversity and engage new actors to conservation (Kamal et al. 2015; Gooden and Sas-Rolfes 2020). There are many motives for conservationists to support the increased attention to private land (Gooden 2019; Gooden and Sas-Rolfes 2020). On one hand, private lands fulfill many of the same functions as public protected areas, including ecosystem services (such as climate regulation, freshwater supply, water regulation or air quality) and social ones (such as recreation, spiritual and cultural heritage) (Langholz and Lassoie 2001). On the other side, private lands also can provide important ecological functions as corridors and buffer zones for larger protected areas (Willis et al. 2012). In addition, the introduction of new social actors into conservation may increase potential for innovation and entrepreneurship and this can lead to better, more viable, and collaborative decisions (Kerr and Tindale 2004; Moon et al. 2014; Gooden and Sas-Rolfes 2020), while simultaneously the proximity to conservation easements can increase nearby property economic values (Reeves et al. 2018). However, private conservation initiatives may also be controversial, as can be argued that it is a form of privatization of protected areas or commodification of nature conservation. Several uncertainties are derived from the private nature of these private lands. For instance, private land conservation is driven by a variety of influences, such as the voluntary action undertaken by landowners, which is influenced not only by external factors such as financial incentives, but also by personal and psychological factors (Gooden 2019). Top-down approaches to biodiversity conservation on private land have had negative repercussions, with landowners expressing their unwillingness to participate in conservation strategies that provide no benefits for them (Grodzinska-Jurczak and Cent 2011; Kamal et al. 2015). On the other side, most private lands are informally protected (Langholz and Lassoie 2001) and this can promote several future uncertainties regarding the future continuation in a long term. Integrating private land into conservation planning and management is complicated by the nature of landownership and the complex social and economic traits that are interrelated with its current use (Mascia 2003; Raymond and Brown 2011; Kamal et al. 2015).

Our approach can be used to implement transdisciplinary processes where the scientific, public, and policy-making communities work together with the goal of developing private conservation strategies (López-Rodríguez et al. 2017). Although our results refer to a specific case study in the Western U.S., the approach proposed can be easily translated to other areas in the world, because private lands are proliferating both in the developing world and in industrialized countries. In Africa, for example, a long history of game ranches has provided important areas for creating private reserves (Langholz and Lassoie 2001). In other regions in Latin America, private lands are also expanding, such as in Colombia or Brazil, because it represents an alternative to the government’s insufficient management. In addition, recently different governments are establishing private land conservation mechanisms for motivating its implementation (Gooden and Sas-Rolfes 2020). For example, the Chilean government has passed legislation to permit the derecho real, a newly codified conservation property right (ILCN 2016; Gooden and Sas-Rolfes 2020). In Catalonia (Spain), a land stewardship network called Xarxa de Custodia del Territori has made progress to secure legislation enabling land stewardship agreements and tax incentives (Brandehof 2018). All these strategies related to nature conservation on private land are being explored globally from legal prescriptions to financial incentives and participatory site selection approaches (Kamal et al. 2015). The raising of private land around the world imply needs for the implementation of interdisciplinary approaches that allow to secure the protection of ecological, social, and cultural values of land. Specifically, by increasing the collaboration between NGOs, such as land trust organizations, and interdisciplinary scientists, similar research on public areas can help to answer important management questions while developing applied solutions for conservation (Knight et al. 2008; Bennett et al. 2017). While this study provides a case study example on how to apply interdisciplinary research methods to land trust conservation easement efforts, future studies should closely collaborate with conservation organizations throughout the entire research process in order to achieve the best results for conservation management and decision (Graves et al. 2019).

## Conclusion

Conservation easements implemented by land trusts are the fastest growing mechanism for private land conservation in the U.S. The proposed methodological approach to identify future lands for conservation can increase the success of conservation efforts because it not only implements a biophysical assessment of conservation goals, but also incorporates multiple views, visions, and perceptions of local private landowners. We call the urgent need for collaboration between scientists, land trusts or other conservation organizations, local communities, and managers to evaluate and monitor the current and future state of private conservation areas. This transdisciplinary collaboration will lead to a more effective implementation of applied research into on-the-ground management and might facilitate the involvement of key stakeholders in conservation, which might contribute to increase the success of growing private conservation strategies. Acknowledging the future conservation of private land with high ecological value will require a landowner acceptance of conservation goals; thus it poses the need of establishing new incentives and methodologies to make visible benefits from conservation and making it more attractive, acceptable, and plausible framed in ‘win–win’ scenarios. Future research demands new efforts for promoting transdisciplinary scientific approaches focused on strengthening collaboration among the scientific, public, and policy-making communities when developing and implementing new private conservation strategies.

## Electronic supplementary material

Below is the link to the electronic supplementary material.

Supplementary material 1 (PDF 946 kb)
