# Supplementary material for: An interdisciplinary assessment of private conservation areas in the Western United States
Source: Ambio. 2020 Feb 21;50(1):150–62. doi: 10.1007/s13280-020-01323-x (PMC7708591; doi:10.1007/s13280-020-01323-x)

Ambio

Electronic Supplementary Material
This supplementary material has not been peer reviewed.

Title: **An interdisciplinary assessment of private conservation areas in the Western United States**

Authors: Cristina Quintas-Soriano, Dainee M. Gibson, Jodi S. Brandt, María D. López-Rodríguez, Javier Cabello, Pedro A. Aguilera, Antonio J. Castro

Appendix S1. Methodology for mapping conservation targets.

Cultural Heritage

Cultural heritage was mapped to represent recognized historic and cultural value by the Sagebrush Steppe Land Trust. Cultural heritage represents the special or historic features within a landscape that provide a sense of community continuity and understanding within the local environment (Tengberg et al. 2012).

We modeled cultural heritage based on spatial differences in historical trends of land use/land cover (LULC) weighted by data obtained from the social survey. We used the National Land Cover Database and a US Geological Survey topographic map (NLCD; USGS) for defining LULC classes that consisted of urban area, agricultural area, rangeland, and forested land. To create a distribution map of cultural heritage values in the watershed, we first used ArcMap 10.5 to reclassify five land use maps (from 1970, 1992, 2001, 2006, and 2011) to represent a particular land use (1 if it was the land use, 0 if not), and then we added each of these reclassified rasters together with the raster calculator together to create a final map of historical LULC changes for that land use, with 0 indicating that a particular grid cell was never that land use and 5 indicating that the grid cell has always been that land use. This method was repeated for all four land uses from which we had social survey information to create a map of land use change for each land use type. Then, using the social survey data (see section B, Appendix B) that ranked cultural heritage as impacted by different LULC, we weighted each LULC change map by social perception data to obtain a final measurement of cultural heritage. In the social survey, respondents were required to provide how each LULC type would affect various ecosystem services from the ecosystem services panel either positively and/or negatively on a scale from 1-10, with 10 being the largest impact positively and/or negatively. We calculated the average perceived value of LULC impact on cultural heritage, which then was used as a constant to weigh the different land uses for the final map of cultural heritage value.

Finally, using the raster calculator in ArcMap 10.5, cultural heritage was calculated as (3.1 Urban Land Historical Change) + (7.2 Agricultural Land Historical Change) + (4.1 Forest Land Historical Change) + (6.8 Rangeland Historical Change).

Habitat Quality

Habitat Quality is defined as representing critical wildlife habitat to the Sagebrush Steppe Land Trust. Habitat quality can be defined as general terrestrial habitat availability and relative degradation to anthropogenic risks. We used the InVEST Habitat Quality Model to map habitat quality (Sharp et al. 2016). Inputs for the habitat quality model included the most recent land use/land cover (LULC) map (NLCD 2011), anthropogenic threats to habitat

LULC, the sensitivity of habitats to those threats, and access polygons. Following Villamagna et al. (2017), we used USGS GAP Protected Areas Database and their corresponding GAP Code for management to define the access of large areas to human interference, which indicates how likely an area would be affected by a threat. These habitat threats included agricultural lands, low/medium/high urban lands, primary and secondary roads, and trails in the Portneuf watershed. The threats were weighted following methods from Polasky et al. 2011 and Blumstein and Thompson 2015. Following Polasky et al. (2011), we coded the threats as having an exponential decay, in that as you move away in distance from the threat into the habitat, the threat's impact on habitat decreases exponentially.

<u>Trail Density</u>

Recreation was defined as the Sagebrush Steppe Land Trust's recognition of recreational access as important for conservation easements. To represent these recreational areas in the Portneuf watershed, we used trail density as a proxy for recreational potential. To do so, data from recreation areas with access to the Portneuf River were merged with all parcels of public lands (Bureau of Land Management (BLM) and Forest Service (FS) with trail data. To do this, we used the ArcMap 10.5 Merge tool and obtained the total mileage of trails contained within each parcel using the Summary Statistics tool. We then calculated the density of trails in the Portneuf and assigned that value to the original layer of recreational areas.

<u>Scenic Quality</u>

Scenic quality was mapped to represent the conservation goal of the Sagebrush Steppe Land Trust to preserve open space and scenic views. This conservation goal represents the aesthetic value derived from scenic views and proximity to open spaces (Bagstad et al. 2014). For scenic quality, we used the InVEST Scenic Quality Model (Sharp et al. 2016). This model created a viewshed for each of the negative scenic inputs of the model. In the Portneuf watershed, these points included feedlots, landfills/dumps, point source air emission facilities, barren land use, and industrial (high urban) land use. A total of 111 points throughout the Portneuf watershed were included in this analysis. Combining these negative scenic input points with a 30m digital elevation model for the Portneuf watershed, the model created an output in which areas that are in view of the negative input point have a lower scenic quality than areas that cannot see any of the negative input points. The resulting map included all viewsheds for the input points combined with the other areas of the study area that could not view these negative input points.

<u>Water Quality</u>

Water quality is a stated goal of the Sagebrush Steppe Land Trust, and can be defined as the ecological state, or health, of natural water systems (Queiroz et al. 2015). For mapping water

quality, we used the EnviroAtlas database as a proxy (United States Environmental Protection Agency; https://www.epa.gov/). The EnviroAtlas database provided the length of streams (in kilometers) in each of the subwatersheds of about 105 km2 on average that make up the larger Portneuf watershed that are impaired by various water quality indicators. In the Portneuf watershed, these impairments include temperature, metals, biota, and nutrients. Although each of these impairments can create a singular map for water quality, EnviroAtlas also provides the total length of streams that have some sort of water quality impairment within each subwatershed. Using the field calculator in ArcMap 10.5, we divided the length of all stream impairments by the total stream length within each subwatershed of the Portneuf watershed, which provided a proxy for overall water quality in each region (subwatershed) of the larger Portneuf watershed.

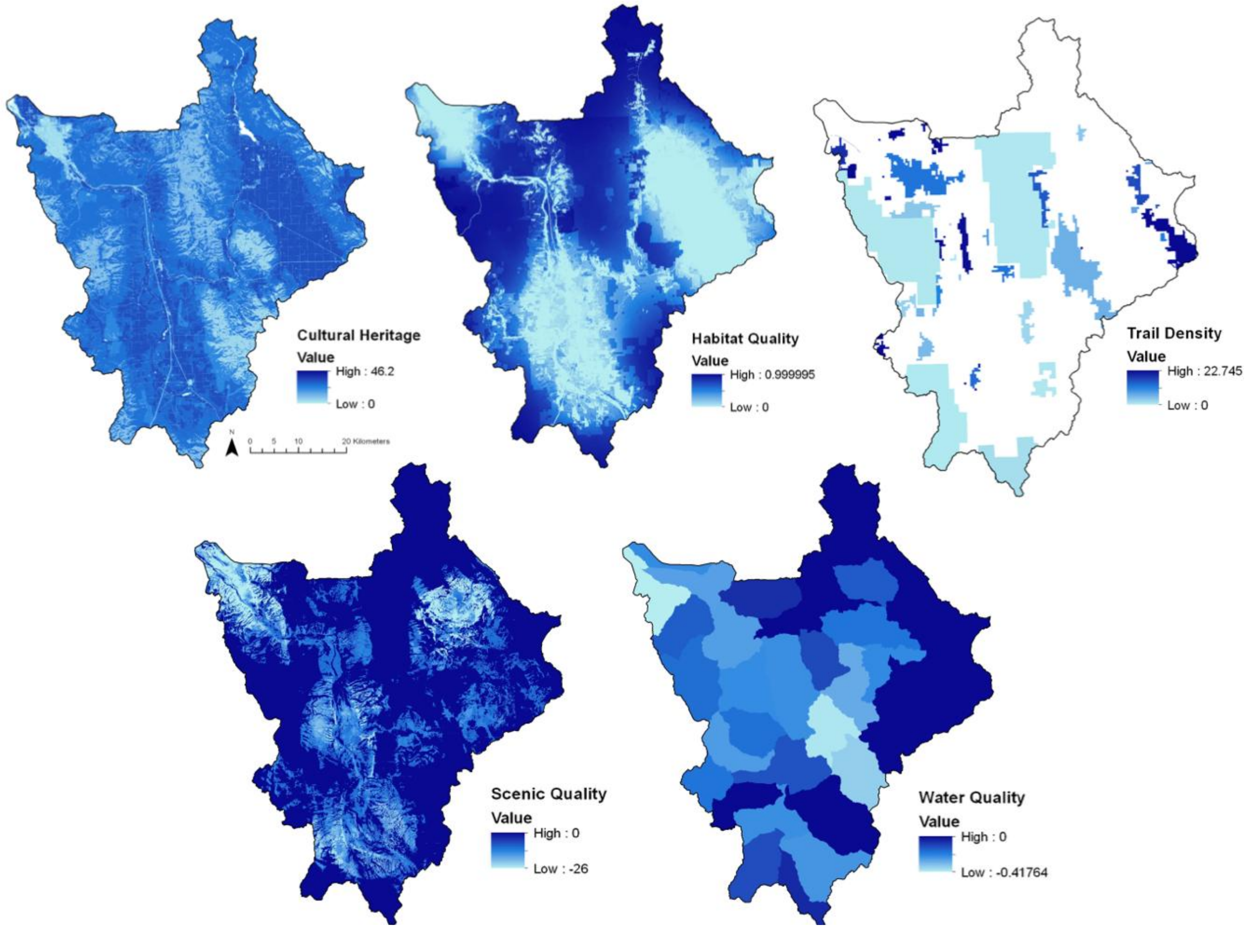

Figure A1. Original conservation target maps in the Portneuf watershed, Idaho.

References:

Bagstad KJ, Semmens DJ, Waage S, Winthrop R. 2013. A comparative assessment of decision-support tools for ecosystem services quantification and valuation. Ecosystem Services 5:27-39.

Polasky S, Nelson E, Pennington D, Johnson KA. 2011. The impact of land-use change on ecosystem services, biodiversity and returns to landowners: A case study in the state of Minnesota. Environmental and Resource Economics 48(2):219-42.

Queiroz C, Meacham M, Richter K, Norström AV, Andersson E, Norberg J, Peterson G. 2015. Mapping bundles of ecosystem services reveals distinct types of multifunctionality within a Swedish landscape. Ambio 44(1):89-101.

Sharp, R., Tallis, H.T., Ricketts, T., Guerry, A.D., Wood, S.A., Chaplin-Kramer, R., Nelson, E., Ennaanay, D., Wolny, S., Olwero, N., Vigerstol, K., Pennington, D., Mendoza, G., Aukema, J., Foster, J., Forrest, J., Cameron, D., Arkema, K., Lonsdorf, E., Kennedy, C., Verutes, G., Kim, C.K., Guannel, G., Papenfus, M., Toft, J., Marsik, M., Bernhardt, J., Griffin, R., Glowinski, K., Chaumont, N., Perelman, A., Lacayo, M. Mandle, L., Hamel, P., Vogl, A.L., Rogers, L., Bierbower, W., Denu, D., and Douglass, J. 2016. InVEST +VERSION+ User's Guide. The Natural Capital Project, Stanford University, University of Minnesota, The Nature Conservancy, and World Wildlife Fund.

Tengberg A, Fredholm S, Eliasson I, Knez I, Saltzman K, Wetterberg O. 2012. Cultural ecosystem services provided by landscapes: Assessment of heritage values and identity. Ecosystem Services 2:14-26.

United States Environmental Protection Agency. EnviroAtlas. Stream length with Total Impairments. Retrieved: June, 26, 2017, from enviroatlas.epa.gov/enviroatlas

Villamagna AM, Mogollón B, Angermeier P. 2017. Inequity in ecosystem service delivery: Socioeconomic gaps in the public-private conservation network. Ecology and Society 22(1).

Appendix S2. Questionnaire administered in the Portneuf River watershed.

DATE .... Nº SURVEY .... INTERVIEWER NAME ....…

Section A: Ecosystem Benefits Perception in the Portneuf Watershed

1. Do you think the Portneuf Watershed provides benefits that contribute to human wellbeing of the region? How many benefits? (Here it's important to explain what we mean by Portneuf Watershed)

- ☐ Very Many
- ☐ Many
- ☐ Few
- ☐ Very little to none

Can you give me examples of some potential benefits? (All they consider)

……………………………………………………………………………………………………

2. From **CONTRIBUTIONS/BENEFITS PANEL**, could you choose what you think are the most important benefits for maintaining wellbeing or quality of life of people living or visiting in the Portneuf Watershed? (Choose only 4) and how do think they have changed? (The same, worse, better, or don´t know) **(SHOW/EXPLAIN CONTRIBUTIONS/BENEFITS PANEL)**

Ecosystem Benefits	***Choose 4** of 11 ES and rank:*<br>*(1) Least important*<br>*(2) Somewhat important*<br>*(3) Very important*<br>*(4) Most important*	*Why are they important? (describe with 1 or 2 words)*	*Using the 4 ESB you chose: In the last 10 years, would you say each has:*<br>*(1) Decreased*<br>*(2) Remained the same*<br>*(3) Increased*<br>*(4) Don´t know*	***Choose** the location on the PW map where this benefit is coming from (write down cell number(s))*
Alternative Energy				
Food from agriculture and livestock				
Freshwater Provision				
Water regulation				
Water quality				
Air quality				
Climate Regulation				
Habitat for species				
Local identity				
Recreation/ Ecotourism				
Cultural Heritage				

Section B: Land Use Perception on Ecosystem Benefits

1. Does land use around the Portneuf Watershed affect the benefits people get from the landscapes?

   □ NO- Why?..□ YES – Why?...

2. Now with more detail, how do you think these land uses affect the benefits that the Portneuf Watershed provides? (**SHOW CONTRIBUTIONS AND LAND USE PANEL**)

Land Uses	*Benefits negatively affected. Choose up to 2, if any, from the services panel and give them a 1 (min) to 10 (max)*	*Benefits positively affected. Choose up to 2, if any, from the services panel and give them a 1 (min) to 10 (max)*
Developed Land		
Agricultural Land		
Rangeland		
Urban and Natural Forest		

Section C: Variables related to environmental behavior

1. Where do you live?
2. Do you belong to any community groups?
3. Are you active in community affairs?
4. How long have you lived here?
5. What geographic location do you identify with most?
6. What year were you born?
7. What is the highest level of school you have completed?
8. What is your profession?
9. What is your annual household income?
10. How would you describe your ethnic background?

Appendix S3. Ecosystem services panel.

DIRECT CONTRIBUTIONS OBTAINED FROM ECOSYSTEMS		INDIRECT CONTRIBUTIONS OBTAINED FROM THE FUNCTIONING OF NATURE		NON-MATERIAL CONTRIBUTIONS THROUGH HUMAN EXPERIENCES	
PROVISIONING		REGULATING		CULTURAL	
CONTRIBUTION	PICTURE	CONTRIBUTION	PICTURE	CONTRIBUTION	PICTURE
**Alternative Energy (hydropower, wind mills, etc.)** Sustainable energy can be produced by water flowing through turbines and wind farms.	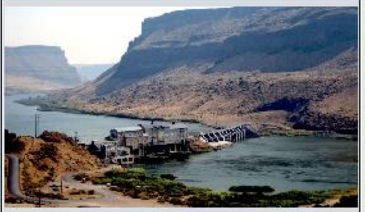	**Water Regulation** Watersheds and ecosystems help regulate the quality and quantity of water available for humans.	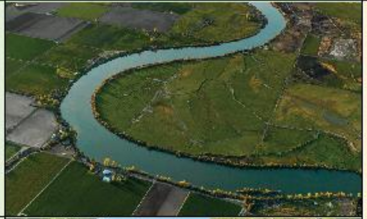	**Local Identity** People in Idaho are very proud of where they come from.	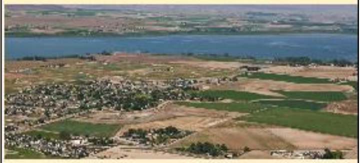
	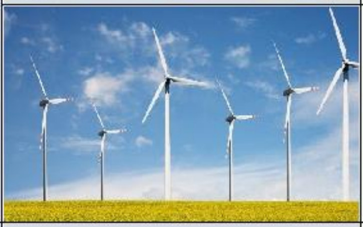	**Water Quality** River species purify water for human use, e.g. mussels filter water and make it clearer.	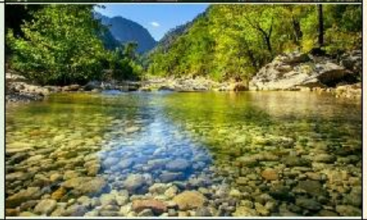		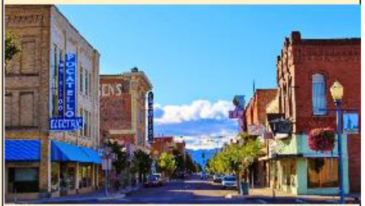
**Food from Agriculture and Livestock** Food products derived from plants, animals, and microbes,	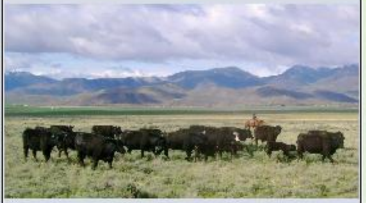	**Air Quality** Forests maintain the quality of air (e.g. the role of ecosystems in the carbon storage).	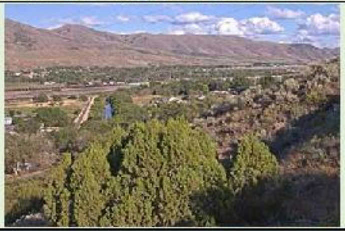	**Recreation/ Ecotourism** Rivers provide experiences through fishing, canoeing, or hiking activities.	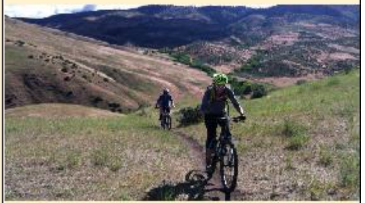
	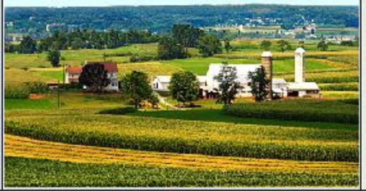	**Climate Regulation** Ecosystems play an important role in climate by either sequestering or emitting greenhouse gases.	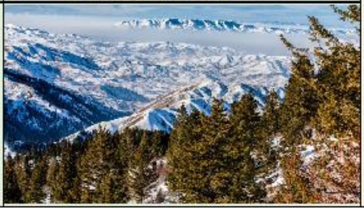		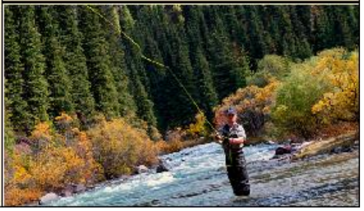

Appendix S4. Principal Component Analysis (PCA) to identify the main explanatory factors of the variability and distribution of the conservation targets across the watershed. Colours correspond with conservation target bundles identified with the cluster analysis. WQ: Water quality; CH: Cultural heritage; SQ: Scenic quality; HQ: Habitat quality; TRAILS: Trails density.

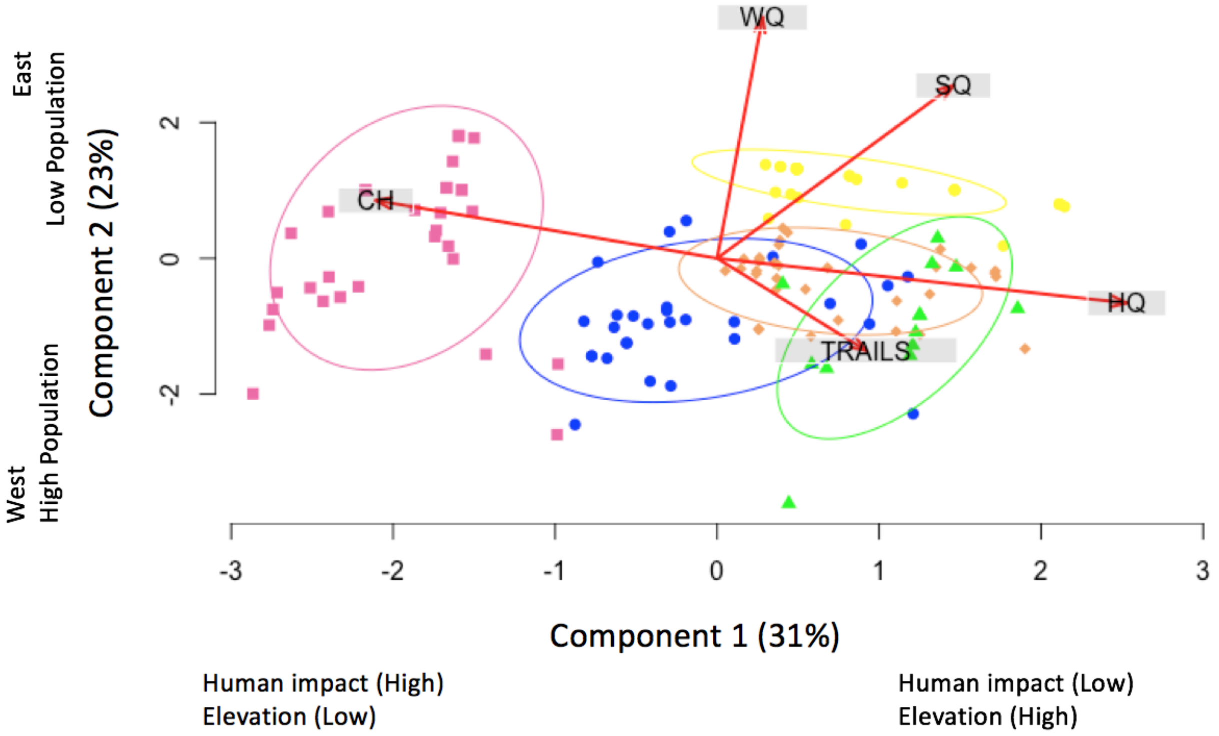

Supplement: Supplementary file 1 — Supplementary material 1 (PDF 946 kb) [file 13280_2020_1323_MOESM1_ESM.pdf]
